# Repetitive injury causes Alzheimer’s disease‐like phenotypes via reactivation of HSV‐1 in a 3D human brain tissue model

**DOI:** 10.1002/alz.089702

**Published:** 2025-01-03

**Authors:** Dana M Cairns, Brooke M Smiley, Jordan A Smiley, Yasaman A Khorsandian, Marilyn A Kelly, Ruth F Itzhaki, David L Kaplan

**Affiliations:** ^1^ Tufts University, Medford, MA USA; ^2^ The University of Manchester, Manchester United Kingdom; ^3^ University of Oxford, Oxford United Kingdom

## Abstract

**Background:**

Millions of people suffer from traumatic brain injury (TBI) annually and many subsequently develop AD‐like characteristics, but the processes occurring in the brain and the reasons for the acquisition of AD‐like dementia are unknown. TBI is the leading cause of mortality in young adults and causes a huge socioeconomic burden. Improving outcomes in these patients would be a significant public health benefit. Evidence indicates that herpes simplex virus type 1 (HSV‐1) in brain of APOE‐e4 carriers confers a strong risk of AD. In a 3D human brain tissue model quiescently infected with HSV‐1, subsequent exposure to other pathogens induces reactivation of the virus via induction of neuroinflammation. We surmised that as TBI also causes neuroinflammation, brain injury might similarly reactivate quiescent HSV‐1.

**Methods:**

Using a mechanical device to initiate closed head injury (CHI), we were able to successfully mimic concussion in 3D tissue engineered constructs consisting of human induced neural stem cells (hiNSCs). We examined the effects of one or more controlled blows to our 3D human brain model in the absence or presence of quiescent HSV‐1 infection, then assessed for downstream AD‐like readouts.

**Results:**

After controlled blows, quiescently‐infected 3D brain tissues showed HSV‐1 reactivation, Abeta and P‐tau production, and gliosis; a phenotype that intensified upon increased repetition of injury. We identified a role for mechanical injury in reactivating HSV‐1 and thus producing AD‐like phenotypes, suggesting that concussion can potentially trigger HSV‐1 to initiate AD pathogenesis.

**Conclusions:**

We suggest that after brain injury from repeated mechanical blows in life, the resulting HSV‐1 reactivation in the brain leads to the development of AD/dementia, i.e., that HSV‐1 is a major cause of AD. We hope to discover ways of alleviating the effects of TBI‐induced HSV‐1 reactivation that ultimately lead to AD, and whether other types of brain damage cause similar effects.

